# Association of invasion-promoting tenascin-C additional domains with breast cancers in young women

**DOI:** 10.1186/bcr2618

**Published:** 2010-08-02

**Authors:** David S Guttery, Rachael A Hancox, Kellie T Mulligan, Simon Hughes, Sinead M Lambe, J Howard Pringle, Rosemary A Walker, J Louise Jones, Jacqueline A Shaw

**Affiliations:** 1 Department of Cancer Studies and Molecular Medicine, University of Leicester, Infirmary Close, Robert Kilpatrick Clinical Sciences Building, Leicester Royal Infirmary, Leicester LE2 7LX, UK; 2 Tumour Biology Laboratory, Cancer Research UK Clinical Cancer Centre, Institute of Cancer Studies, Queen Mary's School of Medicine and Dentistry, Charterhouse Square, London EC1 M 6BQ, UK

## Abstract

**Introduction:**

Tenascin-C (TNC) is a large extracellular matrix glycoprotein that shows prominent stromal expression in many solid tumours. The profile of isoforms expressed differs between cancers and normal breast, with the two additional domains AD1 and AD2 considered to be tumour associated. The aim of the present study was to investigate expression of AD1 and AD2 in normal, benign and malignant breast tissue to determine their relationship with tumour characteristics and to perform *in vitro *functional assays to investigate the role of AD1 in tumour cell invasion and growth.

**Methods:**

Expression of AD1 and AD2 was related to hypoxanthine phosphoribosyltransferase 1 as a housekeeping gene in breast tissue using quantitative RT-PCR, and the results were related to clinicopathological features of the tumours. Constructs overexpressing an AD1-containing isoform (TNC-14/AD1/16) were transiently transfected into breast carcinoma cell lines (MCF-7, T-47 D, ZR-75-1, MDA-MB-231 and GI-101) to assess the effect *in vitro *on invasion and growth. Statistical analysis was performed using a nonparametric Mann-Whitney test for comparison of clinicopathological features with levels of TNC expression and using Jonckheere-Terpstra trend analysis for association of expression with tumour grade.

**Results:**

Quantitative RT-PCR detected AD1 and AD2 mRNA expression in 34.9% and 23.1% of 134 invasive breast carcinomas, respectively. AD1 mRNA was localised by *in situ *hybridisation to tumour epithelial cells, and more predominantly to myoepithelium around associated normal breast ducts. Although not tumour specific, AD1 and AD2 expression was significantly more frequent in carcinomas in younger women (age ≤40 years; *P *< 0.001) and AD1 expression was also associated with oestrogen receptor-negative and grade 3 tumours (*P *< 0.05). AD1 was found to be incorporated into a tumour-specific isoform, not detected in normal tissues. Overexpression of the TNC-14/AD1/16 isoform significantly enhanced tumour cell invasion (*P *< 0.01) and growth (*P *< 0.01) over base levels.

**Conclusions:**

Together these data suggest a highly significant association between AD-containing TNC isoforms and breast cancers in younger women (age ≤40 years), which may have important functional significance *in vivo*.

## Introduction

The role of the stromal microenvironment in modulating breast cancer behaviour is well established [1,2]. A major modulatory component of the stromal environment is the extracellular matrix, and changes in extracellular matrix composition may therefore be expected to be a key factor in determining tumour behaviour. A consistent feature of the stroma around many breast carcinomas is upregulation of the extracellular matrix glycoprotein tenascin-C (TNC) [3-5].

TNC is a complex multifunctional protein that can influence cell behaviour directly and indirectly [6,7]. It has been shown to promote cell migration [8], to inhibit focal adhesion formation [9], to induce cell proliferation [6] and in some cases to act as a cell survival factor [10]. TNC promotes angiogenesis [11] and can induce expression of matrix metalloproteinases [12], which themselves have been implicated in promoting tumour growth and invasion [13].

**Figure 1:**
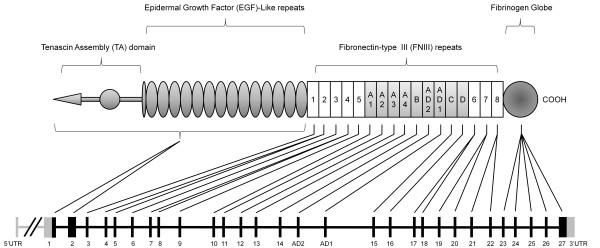
**Schematic representation of tenascin-C**. Protein structure illustrating the N-terminal tenascin assembly domain, the epidermal growth factor (EGF)-like repeat region, the fibronectin type III-like region and the C-terminal fibrinogen-like domain. Exon organisation is shown below (adapted from [25]). Note that the first exon of TNC is not translated and is shown in grey; therefore, exon number 1 in this figure is the first coding exon. UTR, untranslated region.

Structurally TNC comprises a linear arrangement of domains, with a cysteine-rich N-terminus followed by 14.5 epidermal growth factor-like repeats, a region of fibronectin type III-like repeats and a fibrinogen-like domain at the C-terminus (Figure 1) [14]. The structure and size of TNC varies as a result of alternative splicing of domains within the fibronectin type III repeat domain; exons 10 to 16 (domains A1 to A4, B, C and D) can undergo alternative splicing either singly or in combination. A number of biologically active sites have been mapped to the fibronectin type III repeat domain, including recognition sites for cell surface receptors such as integrins [15], cell adhesion molecules of the immunoglobulin superfamily [16], and annexin II [17] as well as sites susceptible to proteolytic cleavage by matrix metalloproteinases [18]. Inclusion or exclusion of different domains in this region can thus generate considerable functional diversity, and up to 22 human splice variants have been identified by RT-PCR analysis [19].

Changes in the profile of TNC isoforms expressed in tumours compared with normal tissue have been described. A switch from the small or truncated form of TNC, which lacks exons 10 to 16, to predominant expression of the large, full-length variant of TNC has been demonstrated in breast, lung and colorectal carcinoma [20-22]. In glioblastoma, upregulation of an isoform containing exon 15 (domain C), which is rarely detected in normal tissues or malignant epithelium, has been demonstrated [23]. We previously identified induction of two intermediate-sized TNC isoforms in breast cancers [24] and showed that these isoforms can enhance tumour cell invasion [25]; a similar change was demonstrated in ovarian carcinomas [26]. Although the precise significance of altered TNC isoform expression is not well understood, it seems likely that different isoforms have distinct biological function, a concept supported in recent studies [25,27].

Two less-studied repeat regions have been identified in human, mouse and avian TNC, termed additional domain (AD) 1 and AD2, inserted between domains B and C [28-30]. Transcripts encoding AD1 have been detected in a variety of cancers [31], whilst inclusion of AD2 has so far been described only in oral squamous carcinomas [28]. Expression of both repeats has been detected in embryonic avian tissue. Interestingly, *in situ *hybridisation demonstrated that TNC-AD1 mRNA is abundant at sites of cell motility and branching morphogenesis, suggesting that inclusion of this repeat may contribute to the migration-promoting effect of TNC [31].

The potential to exploit tumour-specific, alternatively spliced isoforms as therapeutic targets has already been demonstrated [32]. The aims of the present study were therefore to establish whether TNC-AD1 and/or TNC-AD2 domains represent tumour-associated isoforms in breast cancer, to determine whether expression of these isoforms correlates with clinicopathological features and tumour subtypes identified by immunohistochemical profiling [33,34], and to use *in vitro *studies to investigate the functional significance of a TNC isoform containing the AD1 domain to tumour cell behaviour. The results of the study show that TNC-AD domains are significantly associated with breast cancers in younger women (age <40 years), with high grade and with oestrogen receptor (ER)-negative status. Moreover, *in vitro *cell culture models show that, in some cell backgrounds, AD1-containing isoforms promote tumour cell invasion and growth at a higher level than that seen with vector controls and other TNC isoforms.

## Materials and methods

### Tissues and cells

Fresh and/or formalin-fixed tissue was obtained from patients undergoing breast surgery in accordance with Ethics Approval from Leicestershire Research Ethics Committee (06/Q2502/70) and North East London Research Ethics Committee (05/Q0403/199). Since all samples were anonymised there was no requirement from either research ethics committee for informed consent. For isolation of normal cells, tissue was obtained from women undergoing breast reduction surgery who gave informed consent (LNRREC 7054).

Two hundred and four samples were analysed from 155 carcinomas selected based on age (68 infiltrating ductal carcinomas from women aged ≤40 years, 62 infiltrating ductal carcinomas from women aged >40 years, 25 infiltrating lobular carcinomas from women aged >40 years), two cases of ductal carcinoma *in situ*, 14 benign breast lesions including fibroadenoma and fibrocystic change, and 33 normal breast samples from reduction mammoplasty procedures. Tissue was either snap-frozen in liquid nitrogen or routinely processed and paraffin-embedded following formalin fixation. In selected normal samples, tissue was processed and enzymatically digested to yield single-cell populations, and myoepithelial cells, luminal epithelial cells and fibroblast cells were isolated and characterised as described previously [35].

Breast cell lines (MCF-7, ZR-75-1, T47 D, Hs578T, MDA-MB-231, MDA-MB-468, MDA-MB-436, HBL-100 and MCF-10A) were obtained from American Type Culture Collection (Rockville, MD, USA). Most cell lines were maintained in DMEM plus 2 mM L-glutamine and 10% FBS. ZR-75-1 and MDA-MB-436 cell lines were maintained in RPMI supplemented with 10% FBS. The MCF-10A cell line was maintained in a 1:1 mixture of Ham's F12 (Invitrogen Life Science, Carlsbad, CA, USA) and DMEM, 2 mM glutamine supplemented with 5% heat-inactivated horse serum, insulin (10 μg/ml), hydrocortisone (0.5 μg/ml) and epidermal growth factor (20 ng/ml) (all SigmaGenosys, Dorset, UK).

### Transfection of breast carcinoma cell lines

Breast cancer cell lines MCF-7, T-47 D, ZR-75-1, MDA-MB-231 and GI-101 were transiently transfected with a novel TNC-14/AD1/16 construct, generated by PCR ligation using previously generated plasmids (TNC-S, TNC-L, TNC-9/14/16) [25] and a Vector control (empty pCMV Script vector) and FuGene HD transfection reagent (Roche, Welwyn Garden City, UK) according to the manufacturer's protocol. For endogenously TNC-expressing cell lines, co-transfection with the pmaxGFP vector (Lonza, Slough, UK) was also performed to assess transfection efficiency. TNC expression was confirmed using immunohistochemistry for TNC-null cell lines. For all cell lines, expression was also confirmed by quantitative RT-PCR. Transfection efficiencies for each isoform were confirmed by estimating the proportion of cells that were either immunostained positive or were expressing green fluorescent protein (GFP).

### RNA isolation and generation of cDNA

RNA was isolated from frozen tissue for 64 tumour samples, all benign and normal samples and 1 × 10^5 ^cells from each breast cell line using Tri-reagent (Sigma). Seventy tumour tissue samples were available from a previous study in which mRNA was isolated using oligo-dT-linked Dynabeads^® ^(Dynal, Bromborough, UK). Total RNA (1 μg) or all bead-isolated mRNA was reverse transcribed at 42°C for 1 hour using Expand-RT (Boehringer Mannheim, Welwyn Garden City, UK) as described previously [24].

### Quantitative polymerase chain reaction

Owing to limiting amounts of tissue, a nested quantitative PCR approach was devised to amplify TNC exons from frozen tissues. First-round PCR comprised 20 cycles of amplification with primers located in domain 4 (exon 8) and domain 7 (exon 18), to amplify the entire alternative spliced region of TNC (TNC 8/18). Taqman real-time PCR (Applied Biosystems, Foster, CA, USA) was then applied to survey different exons. Inventoried assays were available for the Tenascin-C invariant exon 17/18 boundary (Applied Biosystems Taqman Assay, Hs01115654_m1) and hypoxanthine phosphoribosyltransferase 1 (Applied Biosystems Taqman Assay, Hs99999909_m1) as a housekeeping gene. Primers and probes were developed in-house for AD1 and AD2 (Table 1). For the inventoried Taqman assays, 4 μl cDNA (diluted 1:10) was analysed in a reaction containing 0.5 μl probe, 0.5 μl ultra-pure H_2_O and 5 μl of 2 × Taqman Fast PCR mastermix. For the AD1 and AD2 assays, 3.6 μl cDNA (diluted 1:10) was analysed in a reaction containing 0.2 μl probe, 0.6 μl each primer and 5 μl of 2 × Taqman Fast PCR mastermix.

**Table 1:** Primer sequences for RT-PCR and asymmetric PCR

Primer	Sequence 5' to 3'
T8 - forward	CAATCCAGCGACCATCAACG
T18 - reverse	CGTCCACAGTTACCATGGAG
T16 - reverse	GTTGTCAACTTCCGGTTCGG
AD1 - forward	TGGTGGAGAACACTGGCTATGAC
AD1 - reverse	GGGATCCCCAGCCAAGGT
AD1-FAM-MGB	CAGTGTGGCAGGAAC
AD2 - forward	GATCACCCCCATGAGACCAT
AD2 - reverse	TGATGACAGAGCTGC GAGACA
AD2-FAM-MGB	TGCTGTCTGTGCCTGG
GAPDH - forward	AGAACATCATCCCTGCCTC
GAPDH - reverse	GCCAAATTCGTTGTCATACC
Actin antisense	TCATCACCATTGGCAATGAG
Actin sense	CTAGAAGCATTTGCGGTGGA
AD1 antisense	GAACCAAAGCCACAGTTGG
AD1 sense	TAATGACAAAGGCAGTGAG

Sense and antisense primers were used to generate single-stranded DNA probes for *in situ *hybridisation.

For all breast cell lines and a subset of the carcinomas (11 carcinomas in women aged ≤40 years and 11 carcinomas in women aged >40 years), AD1, AD2 and exon 17/18 were also assayed directly to enable evaluation of AD1 and AD2 expression relative to total TNC expression (exon 17/18). All quantitative PCR analysis was performed using Step-One qPCR software (Applied Biosystems). The number of cycles necessary to produce a product above background (Ct value) was recorded and, after normalisation to the Ct value for hypoxanthine phosphoribosyltransferase 1, the relative expression was determined with the following formula:

$$\text{Relative expression}=2^{-(\Delta \Delta \text{Ct})}$$

### Determination of TNC transcript expression

The number of TNC transcripts was calculated using a standard curve generated from nested isoform-specific 8/18 PCR products. This was then used to calculate the number of molecules in a known concentration of sample. A log_2 _value was then produced from the mean Ct value and normalised against the mean Ct values for the endogenous controls.

### Sequencing

AD1 amplicons were analysed by DNA sequencing. Products were sequenced from AD1-F and AD1-R primers, respectively (Table 1), using 1 μl Big Dye Terminator reactions (ABI, Beverley, UK) and analysed on an ABI Prism 377 DNA sequencer. Sequence profiles were analysed using the Chromas software (version 2.3; Technelysium Pty, Ltd Eden Prairie, MN, USA).

### Immunohistochemistry

Immunohistochemistry for cytokeratin (CK) 14 (Sigma), CK5/6 (Sigma) and P-cadherin (BD Biosciences Cowley, UK) was performed on 4 μm formalin-fixed, paraffin-embedded (FFPE) serial sections from a subset of 96 breast carcinomas. A standard avidin-biotin complex technique was employed with citrate buffer microwave antigen retrieval for P-cadherin. Normal breast tissue was used as a positive control for all antibodies. Negative controls involved omission of the primary antibody. Sections were scored positive for CK14, CK5/6 or P-cadherin if >10% of the tumour cells were stained. The ER and progesterone receptor were examined in 138 tumour cases, and HER2 examined in 134 cases [36]. All interpretation was carried out by RAW and JLJ.

### Western blotting

Levels of cellular and secreted TNC isoforms were determined by western blotting of transfected cell lysates and serum-free culture media respectively. Cells were transiently transfected and incubated for 24 hours, serum-free media was added and the culture media was collected a further 48 hours after transfection. Protein concentrations were quantified on a Lambda 25 UV/VIS spectrophotometer at 750 nm using the bovine serum albumin protein assay, and equal amounts of protein were loaded onto 6% SDS-PAGE gels and transferred to Hybond ECL nitrocellulose membrane (Amersham Biosciences, Little Chalfont, UK). Membranes were blocked in Tris-buffered saline, 5% milk and 1% Tween for 1 hour and were probed for 1 hour with a rabbit polyclonal TNC antibody (clone H300, recognising all forms of TNC; Santa-Cruz Biotechnology, Santa Cruz, CA, USA). A secondary antibody, donkey anti-rabbit horseradish peroxidase-linked IgG (1:2,000; Amersham Biosciences), was added for 1 hour and blots were detected using an enhanced chemiluminescence detection kit (Amersham Biosciences).

### *In situ *localisation of AD1-containing mRNA

Single-stranded sense and antisense AD1 and β-actin control DNA probes were synthesised using asymmetric PCR [24] with specific primers (Table 1) and incorporation of digoxigenin-11-dUTP. *In situ *hybridisation was carried out on 4 μm de-waxed rehydrated tissue sections as described previously [24]. The optimum probe concentration (200 to 500 ng/ml) was titrated to eliminate background staining, whilst retaining good signal strength. Negative controls used sense probes, RNase pretreatment of tissue sections and omission of the probe in the hybridisation protocol.

### Analysis of tumour cell invasion

Measurement of tumour cell invasion was based on modified Boyden Chamber assays using the Fluoroblok tumour cell invasion system as described previously [37]. To measure the direct effect of TNC isoform expression on tumour cell invasion, transiently transfected cells were placed in the upper chamber of the assay and DMEM containing 10% (v/v) FCS was added to the lower chamber as a chemotactic stimulus - except for the MDA-MB-231 cell line, where DMEM containing 1% (v/v) FCS was used. Assays were monitored in real time up to 48 hours with readings every 2 hours and for a total of six replicates representing three separate transfections. Relative fluorescence to time 0 was used as a measure of invasion for each time point,

### Analysis of tumour cell growth

The direct effects of TNC isoform expression on tumour cell growth were assessed using 4',6-diamidino-2-phenylindole staining of cell nuclei as described previously [38]. Tumour cells were transfected, and cultured for 48 hours in DMEM containing 10% (v/v) FCS. Cells were washed with PBS, fixed in cold acetone/methanol (1:1) for 2 minutes and washed twice with PBS. Cells were then stained using 4',6-diamidino-2-phenylindole (1:20,000) in PBS for 3 minutes and washed twice with PBS. Cell nuclei were examined using a fluorescence microscope and counted using an in-house software program provided by Dr AE Sayan (University of Leicester, UK) [38]. Each experiment was performed for a total of nine replicates representing three separate transfections.

### Statistical analysis

Statistical analysis was performed using SPSS 16 (SPSS Inc, Chicago, Illinois, USA). Relationships between factors were measured using the nonparametric Mann-Whitney test, except for grade where Jonckheere-Terpstra trend analysis was applied. All tests were two-sided and *P *< 0.05 was considered significant. For functional assays, two-way analysis of variance was performed.

## Results

### Expression of TNC-AD1 and TNC-AD2 in breast cell lines and isolated cell populations

Nine breast cell lines and isolated normal breast myoepithelial cells and fibroblast cells were screened for expression of TNC 17/18 (an invariant exon boundary, present in all TNC splice variants), AD1 and AD2 by real-time quantitative PCR. TNC 17/18, AD1 and AD2 were absent from ER-positive MCF-7, ZR-75-1, and T-47 D cells (Table 2). Of the AD1-positive cell lines, MDA-MB-436, HBL-100 and primary normal myoepithelial cells expressed the highest levels (>400,000 molecules per 1 μg RNA). All AD1-positive cell lines and isolated breast cell populations also expressed AD2, but at lower levels. When AD1 and AD2 expression was related to levels of total TNC expression, HS578T, HBL100 and primary myoepithelial cells showed the highest percentages of AD1 (>25% total TNC) and HS578T and MCF10A cells showed the highest AD2 percentages (>5% total TNC).

**Table 2:** Relative expression of tenascin-C, AD1 and AD2 in cell lines and isolated normal breast cells

	**Number of TNC transcripts (×10**^**3**^**)**			% of total TNC transcripts	
		
Cell line	Total TNC	AD1	AD2	AD1	AD2
ZR-75-1	0	0	0	0	0
T-47D	0	0	0	0	0
MCF-7	0	0	0	0	0
Hs578T	162.651	41.432	14.574	25.47	8.96
MDA-MB-231	1,293.947	44.383	23.821	3.43	1.84
MDA-MB-436	8,409.526	479.824	55.735	5.71	0.66
MDA-MB-468	984.727	89.74	28.784	9.11	2.92
GI-101	1,529.834	143.854	8.433	9.40	0.55
HBL-100	15,017.045	6,671.261	483.212	44.42	3.22
MCF-10A	91.37	7.399	6.933	8.10	7.59
Primary MEC	2,925.394	1,863.143	142.39	63.69	4.87
Primary fibroblasts	5398.821	87.867	98.044	1.63	1.82

Percentage expression of additional domain (AD) 1 and AD2 relative to the invariant exon 17/18 boundary, as a marker of total tenascin-C (TNC) expression, normalised to the hypoxanthine phosphoribosyltransferase 1 control in cell lines and isolated normal breast cells. MEC, myoepithelial cells.

### Expression of TNC-AD1 and TNC-AD2 in breast tissues

RNA was successfully isolated from 134 carcinomas, all 14 benign breast tissue samples and 33 normal breast tissue samples, demonstrated by detection of GAPDH by manual PCR. All samples were then analysed by quantitative RT-PCR for expression of the invariant TNC exon 17/18 boundary (total TNC), AD1 and AD2. Total TNC was detected in all samples apart from two of the carcinomas. AD1 expression was demonstrated in 42 (31%) carcinomas, five benign breast tissues and 12 normal breast tissues (eight of which were from women aged <40 years). TNC-AD2 was less frequent than TNC-AD1, being detected in 31 (23.1%) carcinomas, four benign breast tissue samples and four normal breast samples. Nineteen carcinomas, one benign sample and one normal sample expressed both AD1 and AD2.

### TNC-AD1 and TNC-AD2 expression in relation to clinicopathological features of carcinomas

**Figure 2:**
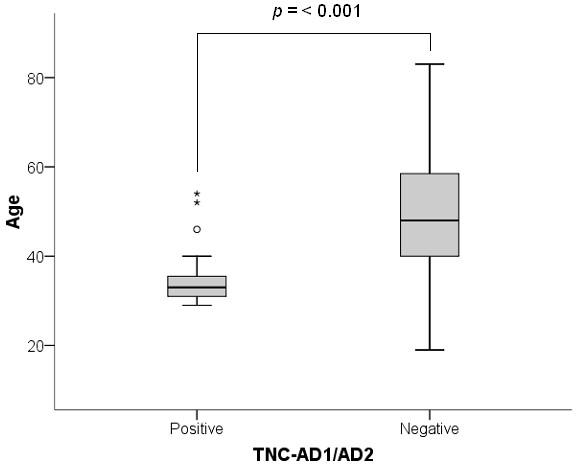
**Distribution of additional domain-positive and additional domain-negative breast carcinomas in relation to patient age**. Number of carcinomas positive or negative for both additional domain AD (1) and AD2. Box contains cases within the lower and upper quartiles, and the line indicates median age. The whiskers connect the youngest and oldest patients that are not outliers; o, outliers; *, extreme values, defined as more than three box-lengths from the box. TNC, tenascin-C.

When quantitative data were compared with clinicopathological features of the carcinomas, expression of AD1 was significantly associated with grade 3 tumours, ER-negative status and with younger patient age (≤40 years) (Table 3). AD2 expression was also significantly associated with younger patient age (Table 3). When the data were stratified by presence of exons only, 19 carcinomas were positive for both AD1 and AD2 - 16 of these were from women aged ≤40 years (*P *< 0.001; Figure 2), and the highest frequency of TNC-AD1-positive and TNC-AD2-positive tumours was seen in women aged between 31 and 35 years. Neither AD1 nor AD2 showed any association with lymph node status.

**Table 3:** Relationship between expression of total TNC, TNC-AD1 and TNC-AD2 and clinicopathological features of tumours

Clinicopathological feature		Total TNC		TNC-AD1		TNC-AD2	
				
	*n*	Mean Ct (95% CI)	*P*	Mean Ct (95% CI)	*P*	Mean Ct (95% CI)	*P*
Age							
≤40 years	47	23.5 (21.8 to 25.3)	0.006	36.6 (35.4 to 37.7)	<0.001	37.7 (36.7 to 38.6)	0.001
>40 years	87	26.5 (25.7 to 27.3)		38.7 (38.1 to 39.3)		39.1 (38.5 to 39.7)	
Tumour type							
IDC	109	25.4 (24.4 to 26.4)	0.554	37.8 (37.2 to 38.4)	0.079	38.7 (38.2 to 39.2)	0.760
ILC	25	25.6 (24.7 to 26.5)		38.6 (37.1 to 40.0)		38.3 (36.5 to 39.9)	
Grade							
I	14	27.6 (25.9 to 29.4)	0.109	39.1 (37.9 to 40.3)	0.017	39.8 (39.3 to 40.2)	0.066
II	63	25.7 (24.6 to 26.7)		38.3 (37.4 to 39.1)		38.7 (37.9 to 39.5)	
III	57	24.7 (23.2 to 26.1)		37.3 (36.4 to 38.2)		38.2 (37.4 to 39.0)	
Lymph node							
Positive	63	25.4 (24.2 to 26.6)	0.802	37.9 (37.2 to 38.8)	0.978	38.8 (38.2 to 39.4)	0.900
Negative	68	25.5 (24.4 to 26.7)		37.9 (37.1 to 38.8)		38.5 (37.7 to 39.3)	
Not known	3						
Oestrogen receptor							
Positive	89	25.6 (24.6 to 26.5)	0.413	38.2 (37.6 to 38.9)	0.011	38.7 (38.1 to 39.3)	0.316
Negative	31	24.1 (22.2 to 26.1)		36.7 (35.3 to 38.0)		38.1 (36.9 to 39.3)	
Not known	14						
Progesterone receptor							
Positive	79	25.4 (24.5 to 26.4)	0.431	37.9 (37.2 to 38.4)	0.441	38.8 (38.2 to 39.3)	0.641
Negative	36	24.5 (22.6 to 26.4)		37.6 (36.4 to 38.7)		38.1 (36.8 to 39.5)	
Not known	19						
Her-2							
Positive	17	23.3 (20.5 to 26.2)	0.232	37.6 (35.9 to 39.3)	0.639	38.2 (36.6 to 39.8)	0.452
Negative	81	25.1 (24.2 to 26.1)		37.8 (37.0 to 38.6)		38.7 (38.0 to 39.4)	
Not known	36						
Basal 1							
Positive	23	24.3 (22.2 to 26.4)	0.605	36.9 (35.3 to 38.6)	0.170	38.4 (37.2 to 39.7)	0.746
Negative	109	25.4 (24.5 to 26.3)		38.0 (37.4 to 38.7)		38.6 (37.9 to 39.1)	
Not known	2						
Basal 2							
Positive	22	25.8 (23.1 to 28.4)	0.103	37.6 (35.9 to 39.4)	0.960	38.5 (37.3 to 39.6)	1.000
Negative	51	23.2 (21.4 to 25.0)		37.6 (36.6 to 38.7)		38.5 (37.7 to 39.3)	
Not known	61						

CI, confidence interval; Ct, number of cycles necessary to produce a product above background; IDC: infiltrating ductal carcinoma; ILC: infiltrating lobular carcinoma; TNC, tenascin-C.

For 22 of the carcinomas (11 carcinomas in women aged ≤40 years, 11 carcinomas in women aged >40 years), AD1, AD2 and exon 17/18 were assayed directly. The total number of TNC molecules and transcripts containing AD1 was greater in carcinomas from younger women (Table 4). It was not possible to statistically analyse AD2 expression since only four samples were positive.

**Table 4:** Tenascin-C transcript levels in carcinomas stratified by age

		Number of molecules			Mean		
			
Age	Sample	Total TNC	TNC-AD1	TNC-AD2	Total TNC	TNC-AD1	TNC-AD2
≤40 years	T1	1,212,265	52,620	0	143,696,985	8,944,007	2,027,925
	T2	99,024,132	1,567,084	0	(*P = *0.005)	(*P = *0.030)	
	T3	2,088,668	150,030	118,774			
	T4	958,188	58,137	0			
	T5	4,701,400	0	0			
	T6	14,962,216	0	0			
	T7	11,837,854	197,415	0			
	T8	339,673	0	0			
	T9	192,103,000	17,251,715	11,618,505			
	T10	1,100,396,396	79,107,073	10,569,893			
	T11	153,043,046	0	0			
>40 years	T12	37,859,646	0	0	4,677,309	25,914	2,211
	T13	208,821	37,702	24,325			
	T14	7,992,309	0	0			
	T15	66,360	7,451	0			
	T16	897,614	0	0			
	T17	73,244	0	0			
	T18	1,206,237	229,110	0			
	T19	168,882	9,045	0			
	T20	276,036	0	0			
	T21	6,970	1,746	0			
	T22	2,694,279	0	0			

Normalised expression of tenascin-C (TNC) molecules in carcinomas from women aged ≤40 years and >40 years. Mann-Whitney tests were used to calculate *P *values.

TNC-AD1 and TNC-AD2 expression was also analysed in relation to the tumour subtype, as determined by immunohistochemical profiling [33,34]. Nielsen and colleagues considered that lack of ER and HER2 and presence of epidermal growth factor receptor (HER1) and CK5/6 could identify basal-like carcinomas [34]. Carey and colleagues defined basal-like carcinomas as ER-negative, PR-negative, Her2-negative, CK5/6-positive and/or CK14-positive [33]. There has been debate as to whether triple-negative cancers (that is, ER-negative, PR-negative, HER2-negative) are the same as basal-like tumours [39]. Rakha and colleagues have recently proposed that CK5/6 and/or CK14 can be used to define basal-like carcinomas irrespective of the expression of the other markers [40]. We therefore chose to examine a range of putative basal markers (ER, PR, HER2, CK5/6, CK14, and P-cadherin [41]) and compared the expression of these with AD1and AD2 expression.

Immunohistochemical profiling and TNC-AD1/AD2 status was known in 132 cases (59 women aged ≤40 years and 73 women aged >40 years) for basal phenotype 1 defined as triple-negative tumours, and in 73 cases (44 women aged ≤40 years and 29 women aged >40 years) for basal phenotype 2 defined as CK5/6-positive and/or CK14-positive tumours. There was no significant relationship between TNC-AD1/AD2 status and either basal phenotype (Table 3). There was also no relationship between TNC-AD1/AD2 status and either HER-2 or P-cadherin expression (data not shown).

### The AD1 domain is part of novel intermediate-sized TNC isoforms

**Figure 3:**
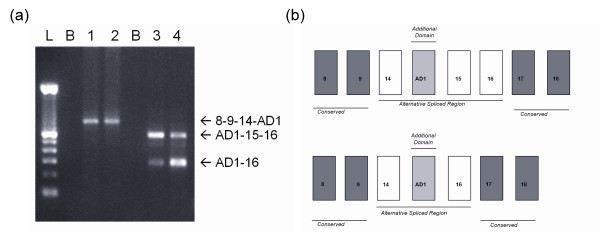
**Identification of two distinct additional domain-1-containing tenascin-C isoforms**. **(a) **RT-PCR analysis in two breast cancers using T8-F and AD1-R primers (Tracks 1 and 2) and using AD1-F and T16-R primers (Tracks 3 and 4). B, blank lane; L, ladder. **(b) **Two alternatively spliced isoforms identified by sequencing, shown schematically.

Sequencing was carried out on two tumour samples that showed expression of two different-sized AD1-containing isoforms (Figure 3a), the largest of which was tumour specific. Blast sequence analysis confirmed that AD1 was located within one of two different intermediate-sized TNC transcripts between exons 9/14 and either exons 15/16 or exon 16 alone (Figure 3b; sequence of AD1 [GenBank:EU295718]) [42]. Neither isoform contained AD2. Analysis of normal breast cell populations isolated from reduction mammoplasties demonstrated a single-isoform TNC-14/AD1/16 in myoepithelial cell populations from five separate donors and in five out of 12 fibroblast populations.

### TNC-AD1 is derived largely from epithelial cells

**Figure 4:**
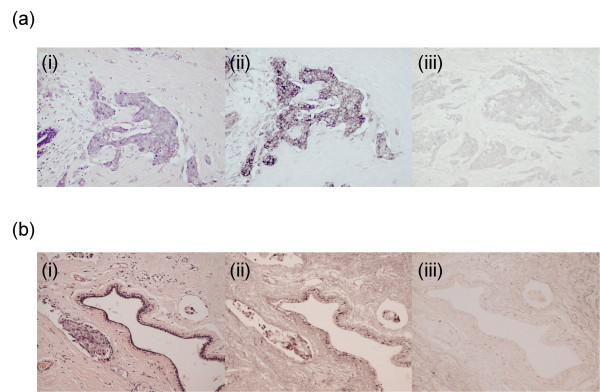
***In situ *localisation of tenascin-C additional domain 1 to tumour cells and normal myoepithelial cells**. **(a) **An example of infiltrating ductal carcinoma positive for tenascin-C additional domain 1 (TNC-AD1) by RT-PCR: (i) H & E; (ii) *in situ *hybridisation using antisense probe to the TNC-AD1 domain, demonstrating signal in tumour cells; (iii) ISH using TNC-AD1 sense probe as a negative control. **(b) **A normal breast duct with adjacent tumour cells within a lymphovascular space: (i) H & E; (ii) ISH with antisense probe to TNC-AD1 showing signal in scattered myoepithelial cells of normal ducts as well as localised to tumour cells; (iii) ISH with TNC-AD1 sense probe as a negative control.

*In situ *hybridisation was carried out on eight breast carcinomas with known TNC-AD1 status using TNC-AD1 probes and β-actin as a control (Figure 4). Specificity of signal was established using sense probes, which were consistently negative. Two carcinomas, which were negative for TNC-AD1 by quantitative RT-PCR, were also negative by *in situ *hybridisation. In six TNC-AD1-positive breast carcinomas, the TNC-AD1 antisense probe localised to tumour cells and not to stromal cells (Figure 4a); and in the associated myoepithelial cells around large histologically normal ducts, there was a lower level of expression detected (Figure 4b).

### Direct effects of TNC isoforms on invasion and growth

**Figure 5:**
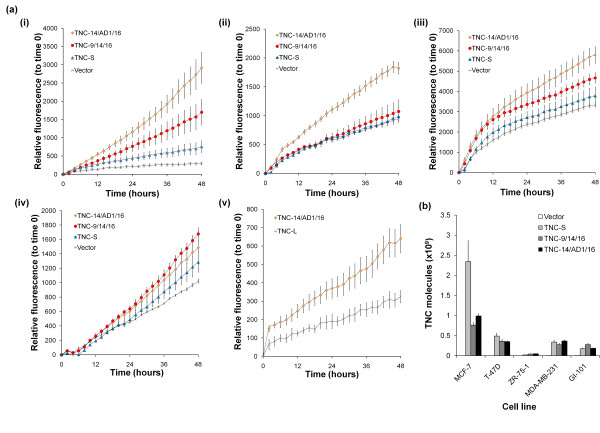
**Direct effects of tenascin-C isoform expression on tumour cell invasion**. **(a) **Monitoring tumour cell invasion in real time using a modified Boyden chamber assay in (i) MCF-7, (ii) T-47 D, (iii) MDA-MB-231 and (iv) GI-101 cell lines transfected with three different TNC isoform constructs and the vector control; and (v) invasion of MCF-7 cells transfected with TNC-L and TNC-14/AD1/16 constructs. In all cases, data points represent the mean of six replicates representing three separate transfections; error bars shown. **(b) **Quantitative RT-PCR of total TNC transcripts (exons 17/18) from cells transfected with TNC constructs.

Although the exon 15-containing isoform (TNC-14/AD1/15/16) was successfully cloned, it was not expressed at the protein level (as determined by western blotting - see Additional file 1) and therefore functional studies focused on the TNC-14/AD1/16 isoform. Transient transfection was used to determine the direct effects of TNC isoforms (TNC-S, TNC-9/14/16, TNC-14/AD1/16) on invasion in the three TNC null cell lines (MCF-7, T-47 D, ZR-75-1) and in two cell lines with endogenous TNC expression (MDA-MB-231 and GI-101) (Figure 5). All cell lines transfected with TNC-S showed no significant increase in invasion compared with vector controls. TNC-14/AD1/16-transfected MCF-7, T-47 D, MDA-MB-231 and GI-101 cells showed increased invasion over vector controls, which became significant with increasing time (Table 5). No significant increase in invasion was evident in the ZR-75-1 cell line.

**Table 5:** Analysis of cell invasion following transient transfection with tenascin-C isoforms

		Cell line				
		
Isoform	Time (hours)	MCF-7	T-47D	MDA-MB-231	GI-101	ZR-75-1
TNC-S	12	NS	NS	NS	NS	NS
	24	NS	NS	NS	NS	NS
	36	NS	NS	NS	NS	NS
	48	NS	NS	NS	NS	NS
TNC-9/14/16	12	NS	NS	NS	NS	NS
	24	NS	NS	NS	NS	NS
	36	*P *<0.05 (NS)	NS (NS)	NS (NS)	*P *< 0.05 (<0.05)	NS (NS)
	48	*P *< 0.001 (<0.05)	NS (NS)	NS (NS)	*P *< 0.001 (0.05)	NS (NS)
TNC-14/AD1/16	12	NS	NS	NS	NS	NS
	24	*P *< 0.05 (NS)	*P *< 0.05 (NS)	*P *< 0.01 (NS)	NS (NS)	NS (NS)
	36	*P *< 0.001 (<0.001)	*P *< 0.001 (<0.001)	*P *< 0.001 (<0.01)	NS (NS)	NS (NS)
	48	*P *< 0.001 (<0.001)	*P *< 0.001 (<0.001)	*P *< 0.001 (<0.001)	*P *< 0.01 (NS)	NS (NS)

Significance at 12-hour intervals relative to vector-only controls (relative to TNC-S). NS, not significant.

TNC-9/14/16-transfected MCF-7 and GI-101 cells also showed increased invasion over vector controls with time. No significant increase in invasion was observed in T-47 D, ZR-75-1 or MDA-MB-231 cell lines (Figure 5 and Table 5). A similar trend in invasion was observed when compared with TNC-S; however, significance appeared at a later time point (Table 5). In MCF-7 cells we also compared mean invasion induced by TNC-14/AD1/16 and TNC-L (this isoform contains all of the exons in the alternatively spliced region, with the exception of AD1 and AD2). TNC-14/AD1/16 significantly enhanced tumour cell invasion over cells expressing TNC-L (*P *< 0.001) (Figure 5a:v). Quantitative RT-PCR analysis of transfected TNC expression showed equivalent levels between constructs in all cell lines - except in MCF-7 cells, which showed TNC-S to be expressed at a higher level (Figure 5b).

**Figure 6:**
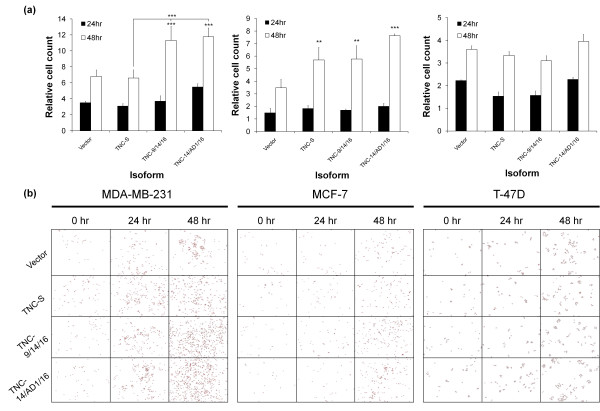
**Direct effect of tenascin-C isoform expression on tumour cell growth**. **(a) **Growth of MCF-7, MDA-MB-231 and T-47 D cell lines transfected with three different tenascin-C (TNC) constructs and a vector control. Bars represent the mean relative cell count compared with 0 hours, with each assay performed three times in triplicate; error bars shown. **(b) **Images of cells transfected with three different TNC constructs. Images were taken using a 4× magnification in exactly the same position in each well. **P < 0.01, ***P < 0.001.

Transient transfection was also used to determine the direct effects of TNC isoforms (TNC-S, TNC-9/14/16, TNC-14/AD1/16) on cell growth in MCF-7, T-47 D and MDA-MB-231 cells. A significant increase in cell number was observed 48 hours post transfection in both MCF-7 and MDA-MB-231 cells overexpressing TNC-9/14/16 and TNC-14/AD1/16 (*P *< 0.01 and *P *< 0.001, respectively, for MCF-7 cells; *P *< 0.001 for both isoforms in MDA-MB-231 cells) compared with the vector control. Furthermore, a significant increase was observed in MCF-7 cells overexpressing TNC-S (*P *< 0.01). No significant increase was observed in MDA-MB-231 cells overexpressing TNC-S. MDA-MB-231 cells overexpressing TNC-9/14/16 and TNC-14/AD1/16, however, showed a significant increase in cell numbers compared with TNC-S (*P *< 0.001 for both). No significant differences were found between isoforms and vector controls for T-47 D cells (Figure 6).

## Discussion

The TNC isoforms containing AD1 and/or AD2 have been suggested to be tumour specific [28,31], and TNC-AD1 may contribute to a motility-promoting environment [30]. The present study has shown that expression of only one AD1-containing variant (TNC-14/AD1/15/16) was cancer specific, but higher mRNA levels of all AD1 and AD2 variants were associated with high-grade, ER-negative breast cancers, and cancers arising in younger women. Detection of AD2-containing isoforms was less frequent and at lower levels; we therefore focused on determining the functional significance of AD1 variants and showed that the TNC-14/AD1/16 isoform promoted invasion and growth.

The present study analysed a series of breast cancer cell lines and also isolated cell populations from normal breast (*n *> 5). Quantitative RT-PCR demonstrated expression of TNC-AD1 and TNC-AD2 isoforms in all TNC-positive lines, which were also ER-negative and were characterised by aggressive behaviour [43-45]. Analysis of isolated cell populations from normal breast showed that the predominant source of AD1 and AD2 expression is the myoepithelium. Isolated stromal fibroblasts, the source of most TNC isoforms [24], showed only low-level expression that was not detected for AD1 by *in situ *hybridisation. The HBL-100 cell line also showed a high level of expression, and this exhibits myoepithelial cell characteristics [46]. AD1 was not detectable in normal tissues by *in situ *hybridisation; but was detected at a low level in isolated enriched populations of a single normal cell type. For the breast cancers, the cells expressing AD1 were predominantly malignant epithelial cells rather than fibroblasts. Sequencing of the two AD1 variants identified in cancers, with AD1 incorporated into an isoform containing exons 15 plus 16 or exon 16 alone, are distinct from those described by Mighell and colleagues [28]. In their study of oral tissues, AD1-containing isoforms were identified in normal and reactive lesions as well as tumour tissue, with TNC-AD2 expression rare but tumour specific. In the present study, the TNC-14/AD1/15/16 variant was cancer specific as this was not detected in any of the normal tissues or isolated cell populations.

We demonstrated that the TNC-14/AD1/16 isoform significantly increased breast cancer cell invasion and growth. The largest TNC isoform (TNC-L) has been frequently associated with an invasive phenotype [6,9,27], as have two other prominent isoforms containing exons 16 and 14/16 [24,25]. The TNC-14/AD1/16 isoform showed a much greater effect on tumour cell invasion than TNC-L in MCF-7 cells, however, suggesting that these intermediate-sized isoforms may be most biologically relevant. TNC-14/AD1/16 also produced a greater effect than the TNC-9/14/16 isoform, but this was only significant for MCF-7 and T-47 D cells. The fully truncated TNC isoform (TNC-S) showed no significant effect on tumour cell invasion, consistent with previous reports [25].

The TNC-14/AD1/16 isoform was also shown to increase cell growth in MCF-7 cells at a similar level to isoform TNC-9/14/16, supporting our previous data [26]. In cells that endogenously express TNC (that is, MDA-MB-231), however, transfection with TNC-14/AD1/16 had a much greater effect on cell growth. The precise mechanisms leading to increased cell invasion and growth in the breast are unclear. Ruiz and colleagues [47] and Lange and colleagues [48] have associated triggering of glioma cell migration with a simultaneous mechanism of competitive inhibition of syndecan-4 binding of fibronectin and signalling by lysophosphatidic acid and platelet-derived growth factor (reviewed in [49]). Furthermore, TNC has been shown to be susceptible to degradation by matrix metalloproteinases [50,51], which can reveal cryptic sites present in the TNC molecule [52] - of which AD1 could be a potential site. Further work using peptide fragments containing AD1 could help elucidate these functions.

High expression of either AD1-containing or AD2-containing mRNA in breast cancers was significantly associated with young patient age (≤40 years). AD1 expression was also associated with lack of ER and grade 3 tumours, both of which are features of cancers in younger women. These results correlate with findings by Helleman and colleagues, who suggested that high TNC expression could be an indirect marker for a defective ER pathway due to an inverse correlation between TNC mRNA and ER protein expression [53]. Breast carcinomas in young women exhibit a particularly aggressive phenotype compared with tumours arising in postmenopausal women [54-56]. This may in part be attributed to the greater frequency of high-grade tumours in young women [57]. There is evidence that tumours in young women are biologically distinct, however, being associated with poorer survival independent of tumour grade and stage [58] and showing a higher frequency of loss of heterozygosity compared with grade-matched and stage-matched postmenopausal cancers [59]. The association of AD1 and AD2 expression with younger age supports the hypothesis that these tumours are biologically distinct.

Over recent years there has been increasing recognition of a subgroup of breast carcinomas characterised by high levels of expression of genes and proteins normally associated with the myoepithelial or basal cell population of the breast [33,34,36,60]. This basal tumour subtype exhibits a poorer prognosis than other subgroups [60], occurs with high frequency in BRCA1-mutated tumours [36] and occurs more frequently in younger women [60]. To address whether the TNC-AD1 (myoepithelial-associated) and TNC-AD2 isoforms were associated with basal features, we compared TNC-AD1 status with expression of putative basal markers [33,34]. There was no association of TNC-AD1 expression with either the triple-negative or CK 5/6-positive and/or CK14-positive subtypes, or with any of the other putative basal markers. This may be due to relatively small numbers of basal cases available for analysis, but a larger study was precluded by the need for fresh tissue for analysis.

## Conclusions

The present study has shown a highly significant association between expression of TNC-AD1 and TNC-AD2 and carcinomas arising in young women (≤40 years), and TNC-AD1 is sometimes incorporated into a novel tumour-associated TNC isoform not detected in normal tissues. The association of TNC-AD1 expression with tumours arising in young women and also with high-grade and ER-negative tumours make it a plausible target for development of novel therapies. Functional studies showed that the TNC-14/AD1/16 isoform can significantly increase breast cancer cell invasion and growth, to a greater extent than the previously described tumour-associated TNC-9/14/16 isoform. This raises interesting questions regarding the functional significance of TNC isoforms containing the AD domains.

## Abbreviations

AD: additional domain; CK: cytokeratin; Ct: number of cycles necessary to produce a product above background; ER: oestrogen receptor; DMEM: Dulbecco's modified Eagle's medium; FBS: foetal bovine serum; FFPE: formalin-fixed: paraffin-embedded; GFP: green fluorescent protein; H & E: haemotoxylin and eosin; PBS, phosphate buffered saline; PCR: polymerase chain reaction; RT: reverse transcriptase; TNC: tenascin-C; TNC-9/16: tenascin-C with only exon 16 of variable region; TNC-9/14/16: tenascin-C with only exons 14 and 16 of variable region; TNC-14/AD1/16: tenascin-C with only exons 14: AD1 and 16 of variable region; TNC-L: tenascin-C largest splice variant; TNC-S: tenascin-C fully truncated splice variant.

## Competing interests

The authors declare that they have no competing interests.

## Authors' contributions

JHP, JAS and DSG created the TNC isoform expression vectors. DSG carried out quantitative RT-PCR assays, invasion assays, cell growth assays and blotting. RAH carried out extraction of RNA from breast tissue, performed nested RT-PCR assays and extracted RNA from isolated myoepithelial cell and fibroblast populations. KTM and SH performed immunostaining of tumour tissue, which was reviewed by JLJ and RAW. SML isolated primary cells and RNA from breast reductions. JHP, JLJ, JAS, RAH, RAW and DSG conceived the study, participated in its design and coordination, and helped to draft the manuscript. JHP, DSG and JAS performed statistical analysis. All authors read and approved the final manuscript.

## Supplementary Material

Additional file 1 **Western blot analysis of TNC expression in transiently transfected MCF-7 cells**. Figure demonstrating a single species of TNC present in the conditioning media and whole-cell lysate. TNC-S is seen as a band at approximately 200 kDa, with slightly larger bands detected for TNC-B/D (exons 14/16) and TNC-B/AD1/D (exons 14/AD1/16), with TNC-B/AD1/D detected at approximately 270 kDa. Click here for file
