# Correction to: Association of invasion-promoting tenascin-C additional domains with breast cancers in young women

**DOI:** 10.1186/s13058-018-0997-8

**Published:** 2018-08-01

**Authors:** David S. Guttery, Rachael A. Hancox, Kellie T. Mulligan, Simon Hughes, Sinead M. Lambe, J. Howard Pringle, Rosemary A. Walker, J. Louise Jones, Jacqueline A. Shaw

**Affiliations:** 1 Department of Cancer Studies and Molecular Medicine, University of Leicester, Infirmary Close, Robert Kilpatrick Clinical Sciences Building, Leicester Royal Infirmary, Leicester, LE2 7LX UK; 2 Tumour Biology Laboratory, Cancer Research UK Clinical Cancer Centre, Institute of Cancer Studies, Queen Mary’s School of Medicine and Dentistry, Charterhouse Square, London, EC1 M 6BQ UK

## Correction

After the publication of this work [1] an error was noticed in Fig. 6 (b). In the MCF-7/Vector columns, the same image was used accidentally for the 0 h and 24 h time points. Both images were taken from the 0 h time point.
