# Supplementary figures and images for: Association of invasion-promoting tenascin-C additional domains with breast cancers in young women
Source: Breast Cancer Res. 2010 Aug 2;12(4):R57. doi: 10.1186/bcr2618 (PMC2949648; doi:10.1186/bcr2618)

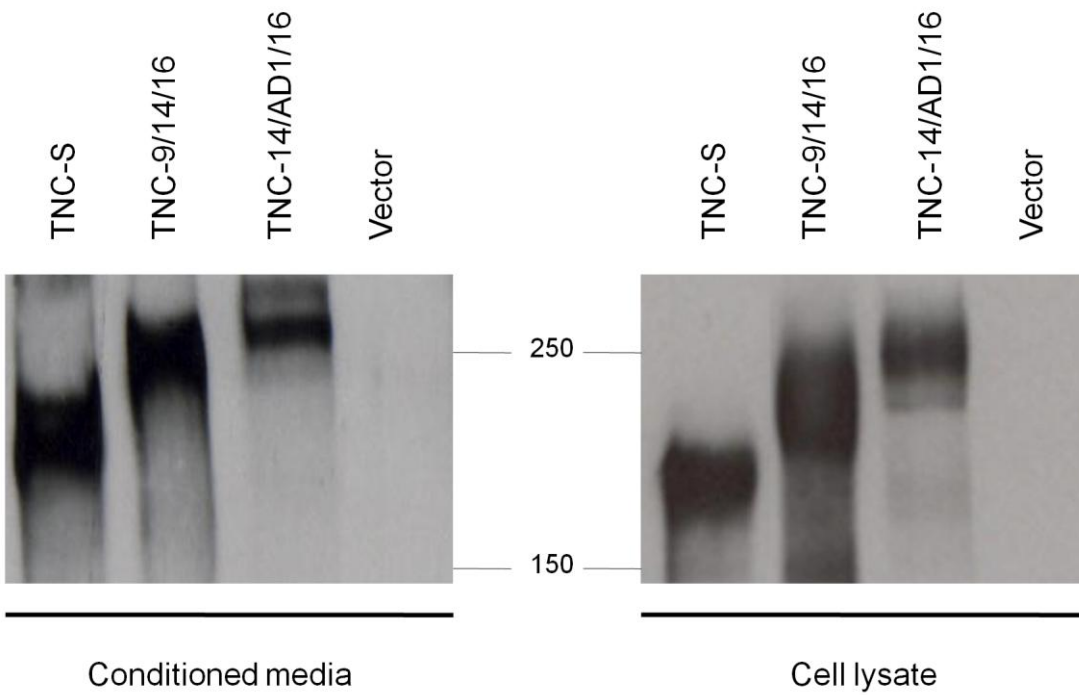

Supplement: Additional file 1 — Western blot analysis of TNC expression in transiently transfected MCF-7 cells. Figure demonstrating a single species of TNC present in the conditioning media and whole-cell lysate. TNC-S is seen as a band at approximately 200 kDa, with slightly larger bands detected for TNC-B/D (exons 14/16) and TNC-B/AD1/D (exons 14/AD1/16), with TNC-B/AD1/D detected at approximately 270 kDa. [file bcr2618-S1.PDF]
